# Operation for Inguinal Aneurism

**Published:** 1836

**Authors:** 


ART. V.—OPERATION FOR INGUINAL ANEURISM. *By Wm. H. Ruan, M. D., of St. Croix.*

The same number of the Journal contains a report of an interesting and difficult operation for aneurism on the right external iliac artery of a mulatto man, 48 years of age, a resident of one of the West India Islands. The swelling in the groin, though small at first, increased rapidly in size until the limb measured five inches more in circumference above the trochanter major than the one on the opposite side. The tumor had a strong pulsation, which spread over an extensive surface, which could be handled without giving any pain. The limb below was much swollen, with considerable venous conjestion, and the entire case afforded but little prospect of a cure even by an operation. An operation however was decided upon, and we will let the surgeon describe it for himself.

"I made the first incision through the skin and subcutaneous cellular substance, commencing about one inch above the superior anterior spinous process of the ilium and an inch and a half on the inside of it, carrying it down with a very slight curve to the right of the patient, and terminating above the upper edge of Poupart's ligament at about half an inch on the outside of the course of the artery; thus making a cut of about three inches in length. The tendon of the external oblique muscle being next divided through the length of the first incision, the fibres of the internal oblique muscle were exposed. These were partially divided at the lower portion of the incision by the knife, and pushed upwards by the finger. I next inserted my finger between the peritoneum and superincumbent transversus abdominis muscle and the remaining fibres of the internal oblique, and with a curved bistoury divided these upwards. By pushing the peritoneum upwards, I readily felt the external iliac artery beating strongly. I experienced some difficulty in separating the fibres of fascia propria of the vessels, so as to disengage a portion of the artery from the continuous vein; but by a little perseverance with the nails of my fore fingers and the handle of a scalpel, I accomplished it; and, with an ordinary aneurismal needle, I then passed a strong ligature around it, and tied it firmly. The pulsation of the tumor, of course, immediately stopped. The ligature consisted of a strong lint thread, doubled and waxed; and was passed under the artery from the out to the inside of it; this mode being found, in the present case, to be the most easy. Care had been taken, during the operation, to disturb the peritoneum as little as possible. No small arteries bled, and the patient did not lose more than a couple of table-spoonsful of blood from a small superficial venous twig which had been wounded, and which retracted and ceased to bleed on being entirely cut across.

The ligature was allowed to hang out at the lower end of the wound the edges of which were kept together by a single stitch and two strips of adhesive plaster. Finally, a firm compress and bandage were applied.

The temperature of the limb rose, immediately after the operation, considerably above that of the opposite side. My prescriptions were a diet of animal food principally, two glasses of wine in the course of the afternoon, and applications of bottles of warm water on each side of the limb through its whole length, and to the sole of the foot."

After the operation, the wound healed speedily without any unpleasant symptoms, and the patient returned to his occupation. W. W.

### Art. V.—Steam and Botanic Journals.

We have recently received a number of Steam and Botanic Journals, containing a variety of rare articles on the medical improvements of the present day. It appears from a Worthington monthly, that two of the professors in the "Reformed College" at that place, have really amputated the leg of a boy whose foot and ankle were much injured by an accident! The case is detailed with all the minuteness of an original operation, and we presume, from the manner in which it is related, the operators anticipate much credit for the skill displayed on the occasion. The professors claim to be teachers in an improved medical institution, and yet they are compelled to add the common names of all the bones and other parts concerned in the operation, that their readers may understand what they are saying, although they are writing for the benefit of the profession! Thus after saying tibia and fibula, terms known to the merest tyro in medicine, they add, "*both bones of the leg;*" after patella, they say, "*or knee pan,*" &c. Ye powers preserve us from such reformed doctors.

We find from the same monthly that the said Botanic Reformers and Steamers have engaged in a violent quarrel, as to which are the real Simon Pures of reform. The Thompsonian Recorder is said to contain "a tissue of wilful, *bare faced, slanders,* and *misrepresentations,*" while the Worthington Monthly, if we may credit the report of the steamers, is no better. The truth is, the days of such reformers are numbered, and their advocates will soon cease to prey upon the ignorant. Still it is not probable that we will be entirely relieved from quackery, for the present sects will no sooner pass away than others, equally notorious, will be warmed into existence by vulgar prejudice. W. W.

